# Impact of nanotechnology on conventional and artificial intelligence-based biosensing strategies for the detection of viruses

**DOI:** 10.1186/s11671-023-03842-4

**Published:** 2023-04-01

**Authors:** Murugan Ramalingam, Abinaya Jaisankar, Lijia Cheng, Sasirekha Krishnan, Liang Lan, Anwarul Hassan, Hilal Turkoglu Sasmazel, Hirokazu Kaji, Hans-Peter Deigner, Jose Luis Pedraz, Hae-Won Kim, Zheng Shi, Giovanna Marrazza

**Affiliations:** 1 School of Basic Medical Sciences, Clinical Medical College & Affiliated Hospital, Chengdu University, Chengdu, 610106 China; 2 Institute of Tissue Regeneration Engineering, Dankook University, Cheonan, 31116 Republic of Korea; 3 Department of Nanobiomedical Science, Dankook University, Cheonan, 31116 Republic of Korea; 4 BK21 NBM Global Research Center for Regenerative Medicine, Dankook University, Cheonan, 31116 Republic of Korea; 5 Mechanobiology Dental Medicine Research Center, Dankook University, Cheonan, 31116 Republic of Korea; 6 UCL Eastman-Korea Dental Medicine Innovation Centre, Dankook University, Cheonan, 31116 South Korea; 7 Department of Metallurgical and Materials Engineering, Faculty of Engineering, Atilim University, 06836 Ankara, Turkey; 8 Centre for Biomaterials, Cellular and Molecular Theranostics, School of Mechanical Engineering, Vellore Institute of Technology, Vellore, 632014 India; 9 Department of Mechanical and Industrial Engineering, Biomedical Research Center, Qatar University, 2713, Doha, Qatar; 10 Department of Biomechanics, Institute of Biomaterials and Bioengineering, Tokyo Medical and Dental University, Tokyo, 101-0062 Japan; 11 Institute of Precision Medicine, Medical and Life Sciences Faculty, Furtwangen University, 78054 Villingen-Schwenningen, Germany; 12 NanoBioCel Group, Laboratory of Pharmaceutics, School of Pharmacy, University of the Basque Country, 01006 Vitoria-Gasteiz, Spain; 13 Biomedical Research Networking Centre in Bioengineering, Biomaterials and Nanomedicine, 28029 Madrid, Spain; 14 Department of Chemistry “Ugo Schiff”, University of Florence, 50019 Sesto Fiorentino, Florence, Italy

**Keywords:** Nanotechnology, Nanomaterials, Artificial intelligence, Nanobiosensors, Viral diagnostics

## Abstract

Recent years have witnessed the emergence of several viruses and other pathogens. Some of these infectious diseases have spread globally, resulting in pandemics. Although biosensors of various types have been utilized for virus detection, their limited sensitivity remains an issue. Therefore, the development of better diagnostic tools that facilitate the more efficient detection of viruses and other pathogens has become important. Nanotechnology has been recognized as a powerful tool for the detection of viruses, and it is expected to change the landscape of virus detection and analysis. Recently, nanomaterials have gained enormous attention for their value in improving biosensor performance owing to their high surface-to-volume ratio and quantum size effects. This article reviews the impact of nanotechnology on the design, development, and performance of sensors for the detection of viruses. Special attention has been paid to nanoscale materials, various types of nanobiosensors, the internet of medical things, and artificial intelligence-based viral diagnostic techniques.

## Introduction

The emergence of new pathogens and the rapid spread of infectious diseases have become a recurring phenomenon and a growing concern across the world [1, 2]. For example, coronavirus disease (COVID-19), a viral infectious disease caused by severe acute respiratory syndrome coronavirus 2 (SARS-CoV-2), has led to the loss of millions of human lives and has decelerated the global economy. The COVID-19 pandemic has also taught us the value of fast and precise on-site pathogen detection and its importance in preventing the spread of infection.

Among the many technological tools available for virus detection, nanomaterials and nanotechnology-assisted biosensors have proven to have advantages owing to their mobility, specificity, and ease of operation by non-experts. Nanotechnology, which is defined as “the science and engineering involved in the design, synthesis, characterization, and application of materials and devices whose smallest functional organization in at least one dimension is on the nanometer scale” [3], has become a powerful platform for the development of various types of nanomaterials and nanoscale devices. These nanomaterials are suitable for the development of highly efficient nanoscale biosensors with enhanced performance and efficacy. In addition, they possess a high surface-to-volume ratio and quantum size effects, offering new possibilities for the detection of various biomolecules and extended limits of detection [4].

**Fig. 1:**
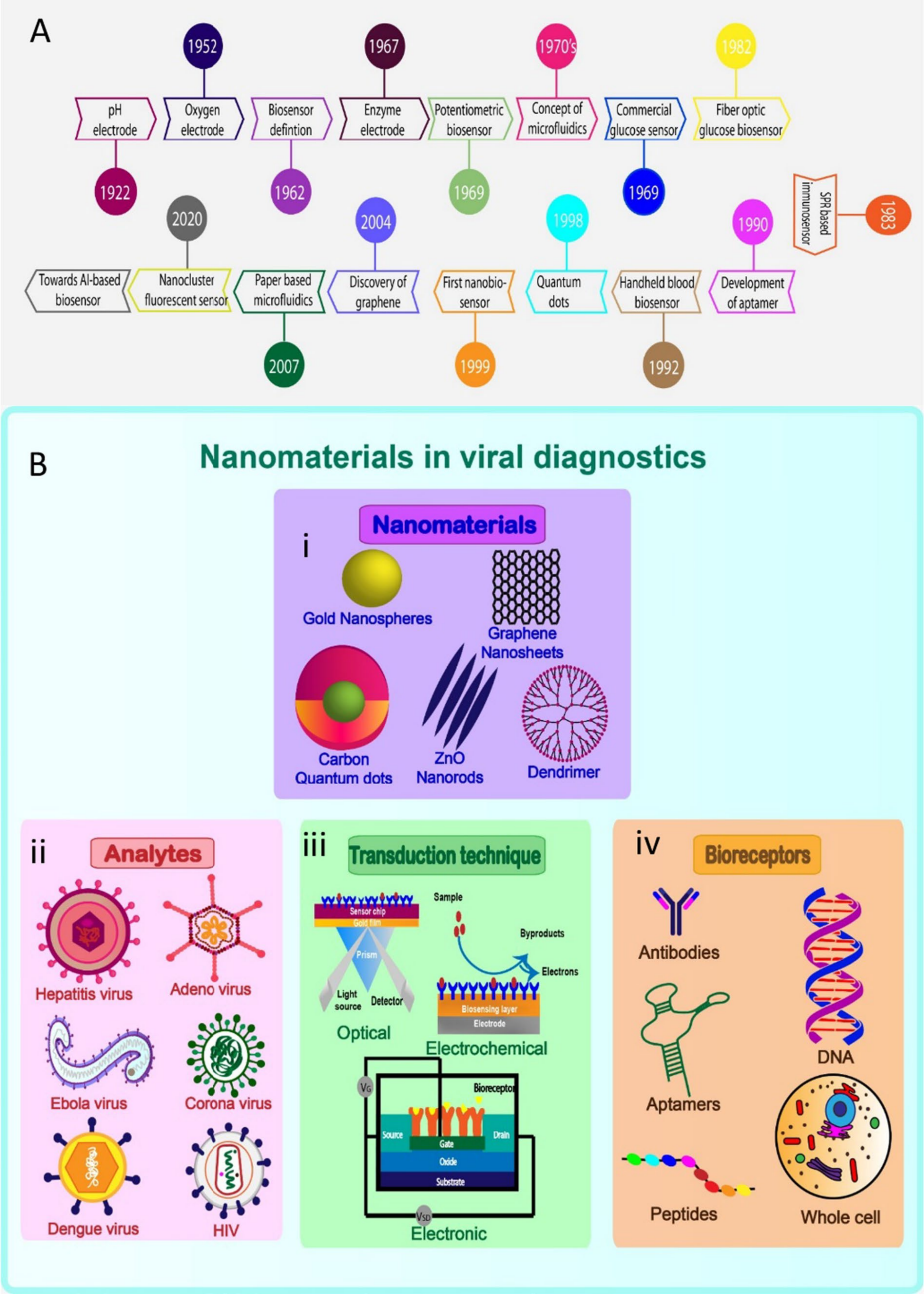
History and the state of the art of Nanobiosensor development: **A** Timeline of the (bio/nano) sensor development from 1920 to 2020. **B** Schematic representation of various forms of nanomaterials, analytes, transduction techniques and the bioreceptors applied for the development of viral diagnostics

Numerous types of nanomaterials, including quantum dots (QDs) and graphene, gold, and silver nanoparticles, have recently been used in the development of sensors [5–7]. Biosensors designed based on nanoscale materials are often called nanobiosensors. These nanotechnology-based biosensors can be broadly classified into four types, namely, the electrochemical [8–11], optical [12–14], thermal [15], and piezoelectric types [16]. Fluorescent nanocluster biosensors, electrochemical nanobiosensors, and microfluidic nanomaterials have all enabled further improvements in detection sensitivity and specificity. Microfluidics, nanotechnology, and biosensors are currently being combined for a wide range of applications [17]. The evolution of nanobiosensors is depicted schematically in Fig. 1A. Although biosensors are being used to detect viruses, several issues remain to be addressed, particularly with respect to their limit of detection and sensitivity. In the recent years, the internet of medical things (IoMT), and artificial intelligence (AI)-based diagnostic techniques play a major role to enable human intervention-free detection of pathogens. It is envisioned that the integration of computer-based technologies, such as IoMT and AI, with conventional diagnostic technologies wound bring out a rapid, precise and user-friendly way of detection systems, which will revolutionize the effectiveness of the biosensors.

Since existing reviews mainly focused on the conventional way of virus detection, the present review cover both conventional and AI-based approaches of virus detection from the nanoscale perspective. With this perspective, this article reviews the impact of nanotechnology on the conventional and AI-based biosensing approaches for the detection of viruses. It discusses the different types of nanomaterials suitable for use in biosensors as well as their key properties, and it highlights the design, development, and application of nanobiosensors in viral diagnostics. Finally, it provides insights into the impact of IoMT and AI on the detection of viruses.

## Nanomaterials in biosensors

Nanomaterials are often used as a transducer that is an integral part of biosensor development and performance. Carbon-based materials such as carbon nanotubes (CNT); graphene; graphene oxide (GO); reduced graphene oxide (rGO) [18]; metallic nanoparticles such as platinum, gold, and silver [18–22]; and metal oxide nanoparticles such as indium tin oxide, titanium dioxide, and QDs [23, 24] have been used in diagnostics to enhance electrode surfaces and substrate functionalities [25]. A schematic representation of nanostructures for viral diagnostics is shown in Fig. 1B.

Gold nanomaterials are important in the field of detection because of their unique electrical and catalytic activity, high biocompatibility, and excellent electron transfer properties [26–28]. Gold nanorods (GNRs) [29], nanoporous gold, gold nanoflowers, gold nanospikes [30], bimetallic gold formations, and gold nanowires are a few forms of gold used for the detection of viruses. GNPs are prepared using a variety of methods, including laser irradiation, chemical reduction, biosynthesis, electrochemical synthesis, and seed growth [31]. GNRs-based sensors have advantages over GNPs, such as a better analytical response, simplicity, higher sensitivity, low sample quantity, label-free, and a lower limit of detection [32]. Nanoporous gold is preferred because its three-dimensional structure provides a high surface area-to-volume ratio, and also due to its robustness, ease of handling, and easily tunable pores. In addition, silver nanoparticles (AgNPs), with their high extinction coefficient and large interfacial surface area that increase electrocatalytic efficiency, are used directly for electrochemical detection [33–35]. Silver nanotriangles (AgNTs) have a stronger plasmonic effect than GNPs, can be fabricated using a simple and inexpensive process, and can be used across large areas [36]. Zinc oxide (ZnO) nanostructures provide faster responses, greater sensitivity, and better electron transfer properties [37].

Graphene, a carbon-based substance with a dense honeycomb crystal structure, has a huge surface area, good mechanical strength, and outstanding electrical conductivity. Thus, it favours electrochemical responses when deposited on an electrode. GO undergoes rapid agglomeration, while rGO can be stored without agglomeration. The oxygen-containing functional groups in rGO are useful for functionalization during the preparation of composites. Graphene QDs (GQDs) possess several favourable characteristics, such as high electron transport and mass diffusion of the analyte, electrocatalyst properties, biocompatibility, and reduced toxicity [38, 39]. CNF possesses a small number of defects, large aspect ratio, low density, high specific modulus, and high electrical conductivity.

Nanocomposites are suitable materials for electrochemical transducer systems owing to their synergistic properties [40]. The elements present in nano-composites maintain their individual features while also imparting novel properties through their combinations [41–44]. The use of different nanostructured materials in the right combination increases the efficiency and sensitivity of detection [45] while significantly reducing non-specific binding towards the analyte. After combination with CNFs, GNPs can get embedded within the sites of defect in the CNFs, increasing the sensitivity and electrochemical activity of the electrode [46, 47]. In yet another nanocomposite formed by the combination of QDs and GNP, the fluorescent properties of QDs were enhanced by the surface plasmon effect of GNPs. Nanomaterials are also well-suited for use as drug delivery systems, potentially paving the way for a new generation of theranostics [48].

## Types of nanobiosensors

Nanobiosensors can be classified into different types, including electrochemical nanobiosensors, optical nanobiosensors, microfluidic nanobiosensors, and nanopore-based nanobiosensors. Each type of nanobiosensor has its own distinct working principle. The different types of nanobiosensors are briefly discussed below:

### Electrochemical nanobiosensors

**Fig. 2:**
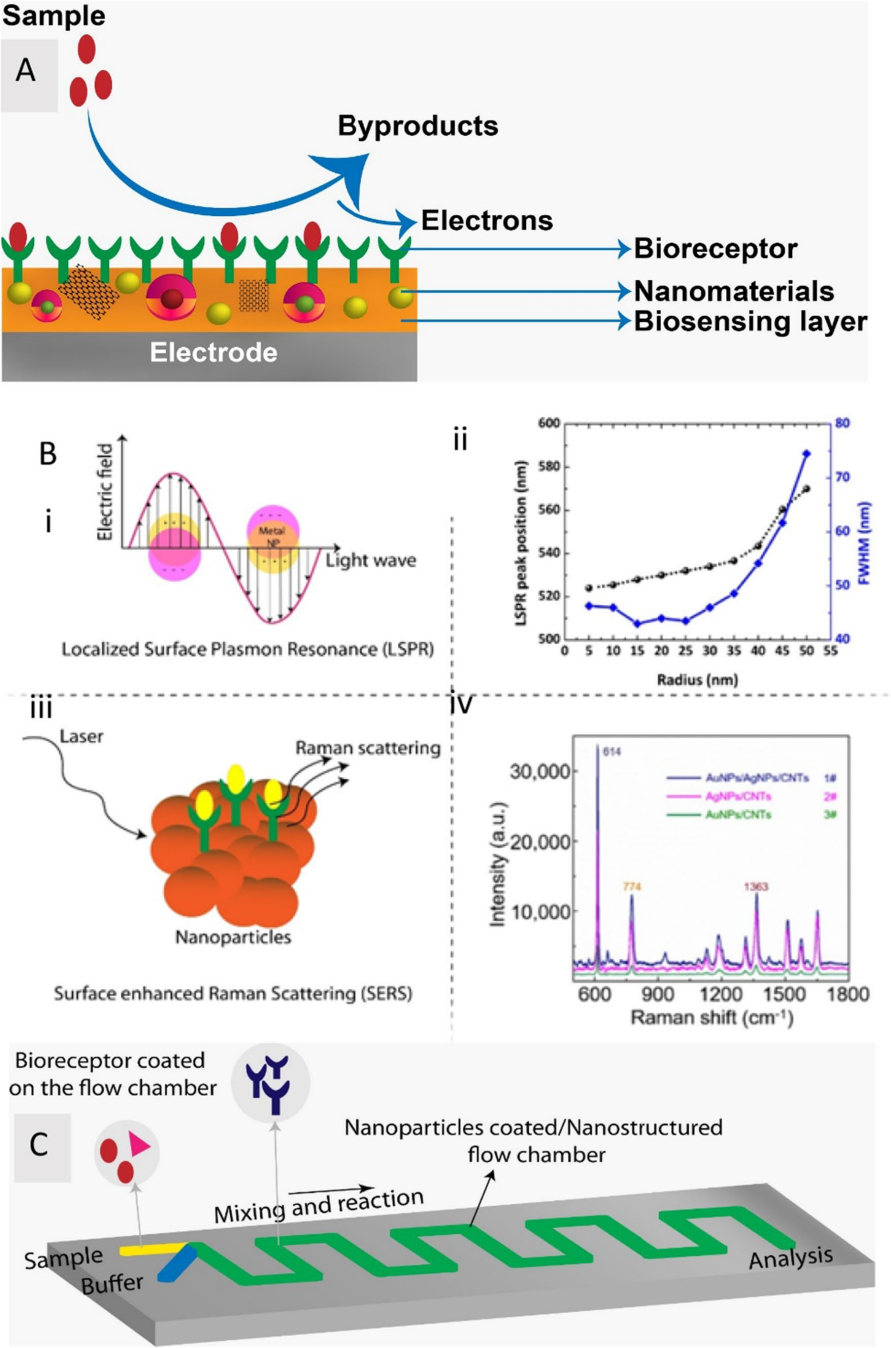
Simple illustrations of Electrochemical, optical and microfluidic nanobiosensors: **A** Schematic illustrates the components of Electrochemical-based nanobiosensor. Here biosensing layer (electrode) is either made using nanomaterials or it is deposited onto it. When the sample interacts with the bioreceptor, by-products and electrons are formed, which is further used for detection. **B** Optical based nanobiosensor: (i) Schematic of the principle of LSPR, (ii) The radius of the nanoparticle is plotted against the LSPR peak resonance wavelength (black spheres) and full width half maximum (blue cubes). Adapted from Farooq et al. [172] under Creative commons Attribution 4.0. (iii) Principle of Surface enhanced Raman scattring (iv) Raman spectra of rhodamine 6 G molecule collected on various film substrates such as Au/AgNP/crossed CNT film (#1), AgNP/crossed CNT film (#2), AuNP/crossed CNT film (#3) Adapted from Wei et al. [173] under Creative commons Attribution 4.0. **C** Illustration of Microfluidic based nanobiosensor. The sample is passed through one inlet and the buffer through the other and then it undergoes mixing and the reaction. It is either fabricated with a nanostructured flow chamber or coated with nanoparticles. Additionally, the flow chamber is coated with bioreceptors. Target-containing samples react with the bioreceptor when they are added, and the reaction products are then analysed in the outlet chamber

Electrochemical sensors require a receptor that can bind to the sample, and an electrode that converts the reaction into a measurable electrical signal (current, voltage, or impedance). The electrode acts as a transducer and as solid support for biomolecule immobilization and electron movement [49]. A high signal-to-noise ratio, ease of use, cost-effectiveness, compactness, high sensitivity, real-time monitoring, rapid reaction, and miniaturization potential are a few of the benefits offered by electrochemical sensing [50–52]. Amperometric [21, 53], potentiometric, impedimetric [54–56], and conductometric sensors are the four types of electrochemical sensors. A variety of biomolecules, including aptamers, proteins, DNA, microorganisms, and toxins, can be detected using these biosensors. The components of electrochemical-based nanobiosensors are shown in Fig. 2A.

Most electrochemical reactions occur near the electrode’s surface, and the electrodes themselves play a significant role in the operation of electrochemical biosensors. The function of the materials utilized to construct the electrode as well as their surface modification and size have a significant impact on their sensing ability [57]. Nanomaterials can be used as carriers for biomolecules to detect targets with high sensitivity because of their high specific surface area. Meanwhile, when nanomaterials are used to modify electrode surfaces, their good conductivity greatly accelerates electron transfer [58]. As a result, platinum, gold, and carbon have attracted serious attention for their use in electrode fabrication given their high conductivity, inertness, biocompatibility, and large surface area.

### Optical nanobiosensors

An optical biosensor generally exploits the optical field’s interaction with a bioreceptor. There are two modes of optical sensing: direct and indirect detection [59]. The indirect detection mode uses optically labelled probes, such as luminescent or fluorescent tags [60], which produce the optical signal. The direct detection mode uses LSPR [61, 62], surface-enhanced raman scattering (SERS) [63, 64], a fiber optic mode, or UV absorption techniques. In this mode, the signal is directly generated by the analyte’s interaction with the optical transducer, eliminating the need for an optically labelled probe. The principle of optical detection enables the development of simple and inexpensive analytical devices with numerous applications in portable nanobiosensor systems that can be efficiently used in remote areas and developing countries [65]. LSPR [66, 67] and SERS [42, 68, 69] have been the most commonly used approaches in optical biosensors and are explained in Fig. 2B. Metal nanoparticles, such as GNPs and AgNPs, possess distinct plasmonic properties that depend on the shape, size, and nature of nanoparticle dispersion on the substrate.

### Microfluidic nanobiosensors

Microfluidics is defined as the “study of flows that are simple or complex, mono or multiphasic, and are circulating in artificial microsystems” [70]. Based on their flow properties, microfluidic biosensors can be classified into various different kinds, including continuous-flow [71], droplet-based [72, 73], digital [74, 75], and paper-based microfluidic nanobiosensors [76, 77]. The small volume of analytes and reagents required, high-throughput nature, portability, real-time detection, low cost, increased specificity and sensitivity, lower energy consumption, and parallel analysis of multiple analytes on one platform are all potential benefits of biosensors that incorporate microfluidics [71]. These sensors can also be integrated with electrochemical and optical transduction systems. Sample preparation, mixing, analysis, and signal readout can be accomplished on a single microfluidic device [78]. A simple microfluidic nanobiosensor is depicted in Fig. 2C. Materials such as glass, silicon, and polymers like polymethylmethacrylate and polydimethylsiloxane are commonly used for the fabrication of these device substrates. The use of nanobiosensors and microfluidic systems to create robust analytical tools represents a significant step forward in the development of self-testing methods that could positively impact healthcare in both developing and developed nations [79].

### Nanopore-based nanobiosensors

**Fig. 3:**
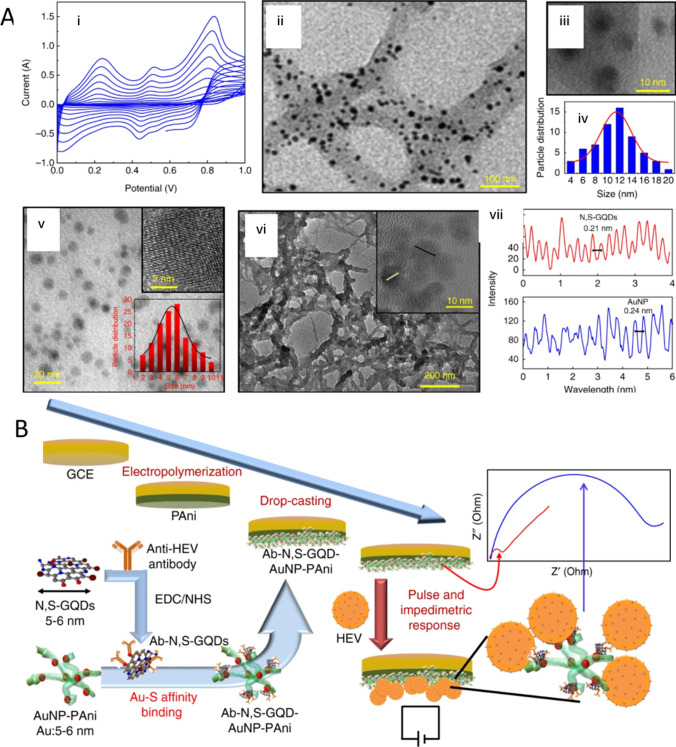
**A** Characterizations of polyaniline deposition, N,S-GQDs and Au-PAni preparation and N,S-GQDs@AuNP-PAni nanocomposites: (i) cyclic voltammograms of electropolymerized PAni film on the GCE electrode, (ii) TEM image of AuNP-PAni nanocomposites, (iii) high magnification of AuNP on the PAni chain, (iv) size distribution of the AuNPs inside the AuNP-PAni nanocomposites, (v) TEM image of N,S-GQDs (inset: HRTEM image of an isolated N,S-GQD and the size distribution), (vi) TEM image of N,S-GQDs@AuNP-PAni nanocomposites (inset: HRTEM of a small area), and (vii) fringe patterns of N,S-GQDs and AuNP from the HRTEM image of N,S-GQDs@AuNP-PAni nanocomposites. **B** Schematic diagram of the Ab-N,S-GQDs@AuNP-PAni nanocomposite-loaded sensor electrode and its electric pulse-induced impedimetric sensing of HEV (Creative Commons Attribution 4.0). Reprinted from [90] with permission from Nature Communications

The nanopore detection of viruses is another rapidly evolving field. While numerous studies have described the application of biological nanopores for viral analyses, the emphasis here is mainly on virus identification via the nanopore-based sequencing of nucleic acids. Nanopores act as biosensors for a whole viral particle, but can also be used to detect and differentiate among distinct viruses in a label-free manner with portable low-cost devices (Fig. 3; for a recent comprehensive review, see Akhtarian et al. [80]). In fact, the use of solid-state nanopores appears to be increasingly successful in the detection of viral particles and diagnosis of viral diseases.

## Application of nanobiosensors in viral diagnostics

Nanobiosensors have tremendous potential in revolutionizing the landscape of diagnostic methods of viral infections. The key applications of different nanobiosensor-based approaches are stated below.

### Electrochemical nanobiosensors in viral diagnostics

Recently, a variety of electrochemical nanobiosensors have been developed for viral detection [81–84]. Screen-printed electrodes (SPE) allow for the production of disposable sensors while also eliminating the disadvantages of traditional electrochemical sensors, such as electrode fouling and the memory effect [52]. Bialobrzeska et al. [85] compared the ability of screen-printed gold electrodes (SPGE) to detect the respiratory syncytial virus with that of reusable glassy carbon disc electrodes (GCDE). In SPGE, the distribution of gold on the electrodes was achieved through 4-aminothiophenol and glutaraldehyde linkers. The detection limit of the SPGE was measured using electrochemical impedance spectroscopy and was found to be three orders of magnitude lower than that of the GCDE. The sensitivity of SPGE was 3.15 × 10^–5^ pfu/mL, whereas the GCDE had a sensitivity of 2.36 × 10^–6^ pfu/mL. The higher sensitivity of SPGE was a result of the increased density of respiratory syncytial antibody coverage on the electrode surface, which was achieved through modification.

A novel electrochemical sensor was developed by Layqah et al. [50] electrodepositing GNPs on a disposable 8-channel carbon electrode array for the multiplexed detection of the Middle East Respiratory syndrome coronavirus (MERS-CoV). In this study, recombinant spike protein S1 was used as a biomarker for the detection of MERS CoV. The carbon electrode array enables multiplexed detection of different coronaviruses. The electrochemical measurements were recorded using square wave voltammetry using ferrocyanide/ferricyanide as a probe. This method was successfully applied to spiked nasal samples, and found to be highly selective, a single-step process, sensitive and accurate in detection. Furthermore, the increased number of electrodes in the chip allows for high-throughput screening, which reduces the overall cost and duration of the test.

A nanoporous gold platform for hepatitis B virus (HBV) detection was developed by Ahangar and Mehrgardi [86]. The porosity of the nanoporous gold was around 70 nm, with a standard deviation of roughly 8.9%, and its detection limit was 5 cycles/10^–14^ mol. Similarly, Rahmati et al. [87] developed a porous 3D N–C@NiCo_2_O_4_ nanowire that was used for detecting the hepatitis C virus (HCV) in conjunction with an HCV core antigen aptamer, yielding a detection limit of 0.16 fg/mL. Because of the porous structure, the active surface area of the nanosystem was increased, and the hydrothermal method was used to synthesize nanowires based on the morphology of sea urchins.

Nanocomposites, especially carbon-based materials combined with nanoparticles, are excellent materials for the fabrication of electrodes for electrochemical detection. Gold electrodes covered with a GO polymer composite can be used as sensors for the detection of dengue virus using electrochemical impedance spectroscopy, as reported by Navakul et al. [88]. In this study, the gold electrodes had a detection limit of 0.12 pfu/mL, and the charge transfer resistance had a linear correlation with virus concentrations ranging from 1 to 2 × 10^3^ pfu/mL DENV. Another nanocomposite was developed by Palomar et al. [89] using multi-walled carbon nanotubes (MWCNT) and a GNP nanocomposite deposited on the gold electrode surface for the detection of dengue virus. The redox peak current intensities were in direct proportion with the electron exchange. Ferri/ferrocyanide (Fe (II/III)) was used as a signal reporter. After the electrodeposition of GNPs, the cathodic current increased from 5.1 × 10^–5^ A to 8.7 × 10^–5^ A. When antibodies were immobilised, the redox peak current of Fe (II/III) decreased from 8.7 × 10^–5^ A to 3.1 × 10^–5^ A. The electrochemically active area of the glass electrode (GE) increased by nearly 30-fold after the incorporation of MWCNT (GE/MWCNT), and it was 10-times higher in GE/MWCNTs/GNP than in GE/MWCNTs. The orientation of the immobilised antibodies affected their activity, but the three-dimensional structure of the porous network of MWCNTs and GNPs facilitated the antibody–toxin reaction. As a result, very minor changes in concentration could be recorded and a very low limit of detection was achieved.

**Fig. 4:**
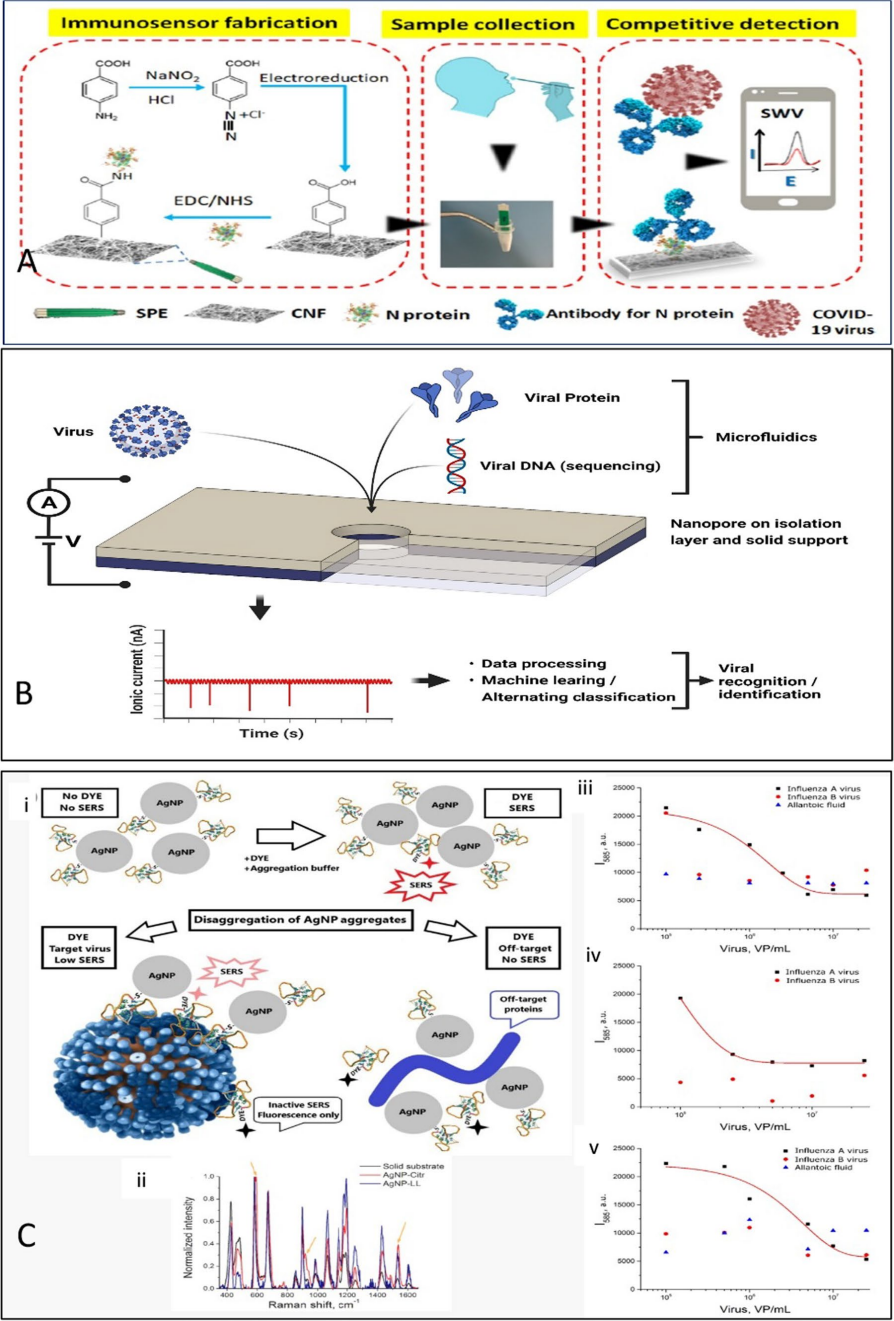
**A** Schematic representing the Cotton-Tipped Electrochemical Immunosensor for COVID-19. Reprinted from [94] with permission from American Chemical Society. **B** Schematic sketch of nanopore biosensing of viruses and viral components. Viral components like proteins can be determined by use of nanopores by applying additional recognition elements like aptamers, viral nucleic acids by (mainly biological) nanopores. Whole virus particles can be identified by similar size nanopores and characteristic signal pattern. Sample concentration and pre-treatment can be integrated into microfluidic units. Electrical signals generated by passing nanopores are processed and can be analyzed by data analysis and statistics software tools for analysing high dimensional data and distinct virus particles (shape, size, rigidity) can be identified. **C** (i) Schematic representation of SERS based aptamer-functionalized AgNP for influenza virus A detection. (ii) SERS spectra of BODIPY FL dye onto substrate, aggregates of AgNP-citrate and AgNP-LL (iii–v) Concentration dependencies of SERS signal intensity of BODIPY FL for different experimental setups. Allantoic fluid was diluted to the same concentration as influenza A virus (Creative Commons Attribution License). Reprinted from [99] with permission from MDPI

A glassy carbon electrode (GCE) surface composed of nitrogen and sulphur co-doped GQDs and gold-embedded polyaniline nanowires with a pulse-triggered electrochemical sensor was developed for the detection of Hepatitis E virus (HEV) [90]. This nanocomposite was created using the interfacial polymerization and self-assembly processes, and it could enhance electrochemical activity and conjugate antibodies with edge carboxylic groups. The preparation of the nanocomposite-loaded electrode and the mechanism of HEV detection are depicted schematically in Fig. 4A. Similarly, Ghanbari et al. [91] developed an electrochemical aptasensor based on GQDs coated onto a GCE for HCV detection. These GQDs allowed better aptamer absorption on the electrode surface, providing a lower detection limit of 3.3 pg/mL.

Al-Douri et al. [92] proposed another nanocomposite combination, an aluminium nanoparticle-doped ZnO nanostructure, for the detection of dengue serotype 2. The nanocomposite was embedded in a p-silicon wafer, into which a silver interdigitated electrode (IDE) was integrated. Consequently, the limit of quantification reached 51.53 μA nM^−1^, with a sensitivity of 55.54 μA nM^−1^ cm^−2^. Another technique involved employing diazonium as a mediator to immobilise the AgNPs that had been functionalized with antibodies on the graphite pencil electrode. The increase in the faradic current with AgNP oxidation was proportional to the NS1 concentration. The proposed method had a detection limit of 0.5 ng/mL [93]. The use of a needle-like quaternary alloy Cu_2_CdSnS_4_ (CCTS) nanostructure deposited on a silicon dioxide (SiO_2_) substrate to detect dengue DNA serotype-2 in real time was described by Odeh et al. [21]. Spin coating and annealing were used to deposit the CCTS nanostructures on the substrate. As a result, a detection sensitivity of 24.2 μA nM^−1^ cm^−2^ and a quantification limit of 56.3 nM were achieved. Eissa et al. [94] established a new type of electrochemical immunosensor with cotton-tipped electrodes, as shown in Fig. 4B. By covering the SPE with absorbing cotton padding, sample collection and detection could be achieved on a single platform. After the CNF and antigens had been deposited on the SPCE, the tapering edge of the electrode was tethered with a bit of cotton fibre to obtain the cotton-tipped electrode. A portable potentiostat connected to a smartphone could be used for electrochemical detection and signal output. To consolidate, a list of nanomaterials employed in recently developed electrochemical nano sensors for virus detection is provided in Table 1.

**Table 1:** Electrochemical detection of various viruses using nanomaterials

Nanomaterial	Nanomaterial size	Analyte	Biorecognition element	Samples	Linear range	Limit of detection	Detection range	Label type	References
GNP	–	Respiratory Syncytial Virus	Anti-RSV antibodies	Virus containing samples	1.0 × 10–1.0 × 10^7^ pfu/mL	1.10 × 10^3^ pfu/mL	0–1.0 × 10^7^ pfu/mL	Label free	[85]
Array of carbon electrodes modified with GNP	50 nm	MERS-CoV	MERS-CoV recombinant spike protein	Spiked nasal sample	0.001–100 ng/mL	1.0 pg/mL	0.001–10,000 ng/mL	Label free	[50]
Nanoporous gold platform	–	Hepatitis B virus	Capture DNA	Human serum plasma	0.4–10 nmol	10 pmol	–	Label	[86]
GO reinforced polymer	500 ± 250 nm	Dengue virus	DNA oligomers	Virus containing sample	1–2 × 10^3^ pfu/mL	0.12 pfu/mL	0–2000 pfu/mL	Label free	[88]
CNF-GNP	CNF-101 nm, GNP-10.1 nm	SARS-CoV-2	Aptamer specific to SARS-CoV-2 RBD domain	Human saliva samples	0.01–64 nM	7 pM	–	Label free	[163]
GQD and gold-embedded polyaniline nanowires	GQD-5–6 nm, GNP-6–14 nm, Polyaniline nanowires-50–70 nm	Hepatitis E virus	Anti-HEV antibody	HEV like particles, human serum and fecal specimens of HEV-infected monkey	1 fg/mL to 100 pg/mL, 10^2^–10^7^ RNA copies/mL	0.8 fg/mL, 96.7 RNA copies/mL	–	Label free	[90]
GQD	–	Hepatitis C virus	Aptamer specific to HCV	Human serum samples	10–70 pg/mL and 70–400 pg/mL	3.3 pg/mL	–	Label free	[91]
AgNP	45–60 nm	Dengue NS1	Dengue NS1 antibody	NS1 spiked human serum samples	3–300 ng/mL	0.5 ng/mL	–	Label	[93]
Aluminium NP doped zinc oxide nanostructures	Aluminium NP-70 nm	Dengue serotype 2	DNA probe	Complementary DNA probe	–	16.9 nM	–	Label free	[92]
Needle-like Cu_2_CdSnS_4_ alloy nanostructure	–	Dengue serotype-2	Dengue-specific DNA probe	–	–	17 nM	100 fM to 10 nM	Label free	[21]
3D N–C@NiCo2O4 NWs	–	Hepatitis C virus	Hepatitis C virus core antigen (HCV) aptamer	Biological samples	0.5 fg/mL to 0.12 pg/mL	0.16 fg/mL	–	–	[87]
Cotton tipped electrode	–	SARS-CoV-2 N protein	SARS-CoV-2 Nucleocapsid Antigen	Nasopharyngeal swabs	1–1000 ng/mL	0.8 pg/mL	–	Label free	[94]

*GNR* Gold nanorods, *DNA* Deoxyribonucleic acid, *GNP* Gold nanoparticles, *RSV* Respiratory syncytial virus, *MERS* Middle east respiratory syndrome coronavirus, *GO* Graphene oxide, *CNF* Carbon nanofiber, *SARS-CoV* Severe acute respiratory syndrome coronavirus, *RBD* Receptor binding domain, *QD* Quantum dots, *HEV* Hepatitis E virus, *HCV* Hepatitis C virus, *AgNP* Silver nanoparticles, *NS1* Non-structural protein 1, *ssDNA* Single stranded DNA, *HIV* Human immunodeficiency virus, *NP* Nanoparticles

### Optical nanobiosensors in viral diagnostics

The chemical stability, versatility, and unique optical propertiaes of GNPs, including LSPR, enable improved optical biosensing. Fu et al. [63] developed a lateral flow assay (LFA) combined with SERS for HIV detection using GNPs conjugated with malachite green isothiocyanate. This colour-based POC diagnostic device could detect concentrations as low as 0.24 pg/mL using a Raman instrument reader. Various nanomaterials such as GNPs, silver nanostructures, and rGO-polyamidoamine (PAMAM) dendrimers have been used in optical sensing for dengue virus detection. Yrad et al. [95] developed a portable strip-based assay using dextrin-capped GNPs for the easier detection of dengue-1 RNA within 20 min. The probes used in this study included a GNP-labelled DNA reporter probe, a dengue-1 specific DNA capture probe (test line), and a complementary DNA probe (control line). Human serum samples were used for sensor validation, and the detection limit was found to be 1.2 × 10^4^ pfu/mL. Subsequently, dengue virus type-2 E protein was detected using SPR by depositing an rGO/PAMAM dendrimer on the substrate. This method provided a sensitivity, dissociation constant, and lower limit of detection of 0.25762 pM, 0.1496 pM, and 0.08 pM, respectively [96].

A chemiluminescent aptasensor that uses magnetic separation and surface-modified GNPs to detect concentrations as low as 0.05 ng/mL has been proposed [97]. Gold, iron oxide, and SiO_2_ NPs were combined to create a nanocomposite that was used to immobilize an HBsAg-specific aptamer for rapid magnetic separation, as well as double functionalized GNPs for signal enhancement. Chen et al. [98] improved the sensor by combining SERS with a three-dimensional nano-popcorn plasmonic substrate containing a gold layer. During the interaction of the DNA aptamer with the influenza A virus subtype H1N1, the Raman peak intensity decreased as the Cy3-labeled aptamer was released from the substrate. Consequently, the mapping image changed colour from bright yellow to dark orange with an increase in virus concentration. For specific recognition of the Influenza A virus, a SERS-based aptasensor with colloidal AgNPs was developed by Gribanyov et al. [99]. The SERS signal increased as the virus interacted with the aptamer. This aptasensor had a dynamic range of 2 × 10^5^–2 × 10^6^ VP/mL and a limit of detection of 2 × 10^5^ VP/mL. A detailed schematic diagram of the SERS-based influenza A virus detection system is shown in Fig. 4C.

An LFA technique was developed by Liu et al. [100] for detecting influenza virus H3 using GNP-coated polystyrene latex microspheres (PLM). Monoclonal antibodies have been used as linkers between GNPs and PLM to prevent non-specific binding because both the particles are negatively charged. Antibodies are more readily conjugated on the surface of GNPs due to their large surface area, and latex microspheres are tunable. When both these agents are used in an appropriate ratio of 16:1 (GNP:PS), the sensitivity increases. This method was used to yield a qualitative detection limit of 1/16 hemagglutination units (HAU) and a quantitative detection limit of about 0.016 HAU. The detection limit of the bare 10-nm GNP-based LFA was 4 HAU, while that of GNP-PS was 1/16 HAU. Hence, it was 64 times more sensitive than the bare 10-nm GNP LFA. A magnetic/plasmonic-assisted fluoroimmunoassay was developed using magnetic-derivatized molybdenum trioxide QDs, which have plasmonic properties. Graphitic carbon nitride QDs were used as the detection probe because of their high fluorescence properties. This technique was tested in clinical samples, and it detected concentrations as low as 45 pfu/mL. Moreover, it had a sensitivity of around 0.25 pg/mL and 0.9 pg/mL in deionized water and human serum containing influenza virus [101]. A GNP–magnetic NP hybrid nanocomposite with CdSeS QDs was prepared by Takemura et al. [102]. The hybrid nanocomposite was used to separate the virus from impurities using a magnetic field induced by the LSPR phenomenon. This phenomenon is significant because it can enhance or quench the fluorescence of QDs, and the intensity of the fluorescence is proportional to the distance between GNPs and QDs. Similarly, GNPs were combined with fluorescent inorganic CdZnSeS/ZnSeS QDs for the development of an LSPR-based nanobiosensor [103]. When the target virus was present, the antibody and antigen created a steric hindrance due to dual antibody anchoring sites in the peptide sequence. This restricted LSPR, resulting in quenching, which was proportional to the concentration of the influenza virus.

Murine norovirus was detected using an optical transduction-based colorimetric method and an ultrasensitive nanozyme aptasensor, which had a detection limit of 3 viruses per assay, equivalent to 30 viruses/mL [104]. In this method, an enzyme that mimics the catalytic activity of GNPs with aptamers was used to convert a colourless substrate into a coloured product. Nanozyme activity was recovered due to the high-affinity AG3 aptamer in the presence of norovirus, causing aptamer desorption. This exposed the catalytic sites on the surface of GNPs, allowing tetramethylbenzidine (TMB) oxidation and the generation of a blue product. The Kd value between the AG3 aptamer and the GNPs was 1.85 × 10^–8^ M, indicating that this aptamer had a higher binding affinity to murine norovirus. A colourimetric aptasensor was also developed for the detection of human papillomavirus (HPV) using GNPs and an RNA aptamer with a hairpin structure against the HPV16 L1 protein. Even in the presence of ppb levels of protein, if salt was added, the GNP and aptamer aggregated. HPV16 L1 protein levels as low as 9.6 ng/mL could be detected [105].

Rodriguez-Moncayo et al. [106] developed a high-throughput, semi-automatic microfluidic device based on antibodies that react against four SARS-CoV-2 proteins: the spike protein, S1 subunit, receptor binding domain, and nucleocapsid protein. With a sensitivity of 95% and specificity of 91%, the device could analyse dozens of samples in parallel at a minimum volume of ~ 6 μL per serum sample. This advancement paved the way for more low-cost and large-scale screening. A single-step colourimetric nanobiosensor in which GNPs interacted with SARS-CoV-2 in nasal and throat swabs was developed by Ventura et al. [107]. In this device, GNPs functionalized with antibodies targeting three surface proteins present in SARS-CoV-2 (spike, envelope, and membrane) at a ratio of 1:1:1 underwent a red shift in the presence of virus particles. To consolidate, the list of nanomaterials employed in the recent development of optical sensors for viral detection is provided in Table 2.

**Table 2:** Optical detection of various viruses using nanomaterials

Nanomaterial	Nanomaterial size	Analyte	Biorecognition element	Method employed	Samples	Linear range	Limit of detection	Detection range	Label type	References
GNP	30–40 nm	HIV-1	DNA Oligonucleotides	SERS based LFA assay	HIV-1 DNA in buffer samples	8–64 ng/mL	0.24 pg/mL	0–64 ng/mL	Label	[63]
GNP	10 nm	Dengue-1 RNA	DENV probe	LFA	Human sera	0.01–0.06 µM	1.2 × 10^4^pfu/mL	–	Label	[95]
Au@Fe_3_O_4_@SiO_2_ NP	–	HBV	HBsAg-specific aptamer	Chemiluminescence	Human serum samples	1–225 ng/mL	0.05 ng/mL	–	Label	[97]
Au/polyethylene naphthalate nano-popcorn substrate	64 nm	Influenza virus A	Influenza virus A specific aptamer	SERS	Clinical samples	–	97 pfu/mL	10–10000 pfu/mL	Label	[98]
GNP	20 nm	Human papillomavirus	RNA aptamer against HPV16 L1 protein	Colorimetric	Clinical samples and vaccine samples	9.6–201.6 ng/mL	9.6 ng/mL	–	Label	[105]
AgNP	4, 10 nm	Influenza virus	Aptamer specific to influenza virus	SERS	Samples containing virus	–	2 × 10^5^ VP/mL	–	Label	[99]
GNP coated polystyrene latex	10 nm, 208 nm	Influenza virus H3 subtype	Anti-influenza virus mAb1	LFA	Virus containing samples diluted in buffer	1/32–32 HAU	0.016 HAU	–	Label	[100]
CdZnSeS/ZnSeS QD and GNP	5 ± 0.5 nm and 28.4 ± 1.5 nm	Influenza virus	Anti-HA antibody (Ab) against influenza virus	LSPR	Serum media	10^−14^–10^−9^ g/mL	65.1 fg/mL	–	–	[103]
rGO-PAMAM Dendrimer	–	Dengue Virus Type 2 E-Protein	Antibodies specific to DENV E-proteins is	SPR	Recombinant dengue virus type 2 E-proteins	–	0.08 pM	0.08–0.5 pM	Label free	[96]
GNP and magnetic NP hybrid nanocomposite with CdSeS QD	GNP-14.8 nm, Magnetic NP-40 nm,	Norovirus	Anti-norovirus genogroup II antibody	LSPR-magnetoimmu-nofluorescence	Clinical samples of feces containing norovirus	–	0.48 pg/mL	1 pg/mL to 5 ng/mL	–	[102]
Plasmonic molybdenum trioxide QD and graphitic carbon nitride QD	3–9 nm	Influenza virus	Antibody against influenza A virus	LSPR-magnetoimmuno-fluorescence	Clinically isolated influenza virus A/Yokohama (110/2009) (H3N2)	–	45 pfu/mL	45–25,000 pfu/mL	–	[101]

*GNP* Gold nanoparticles, *HIV* Human immunodeficiency virus, *DNA* Deoxyribonucleic acid, *SERS* Surface enhanced Raman scattering, *LFA* Lateral flow assay, *GaN* Gallium nitride, *Au* Gold, *Ag* Silver, *SiO*_*2*_ Silicon dioxide, *Hbs* Hepatitis B, *DENV* Dengue virus, *SARS-CoV-2* Severe acute respiratory syndrome corona virus-2, *LSPR* Localised surface plasmon resonance, *NS1* Non-structural protein 1, *RNA* Ribonucleic acid, *HPV* Human papilloma virus, *AgNP* Silver nanoparticles, *QD* Quantum dots, *SPR* Surface plasmon resonance, *rGO* Reduced graphene oxide, *PAMAM* Polyamidoamine, *NP* Nanoparticles, *RBD* Receptor binding domain, *HAU* Hemagglutination unit

### Microfluidic nanobiosensors in viral diagnostics

Lum et al. [108] created an electrochemical impedance-based aptasensor integrated with a microfluidic chip to detect the H5N1 avian influenza virus. The microfluidic chip was covered with gold-coated interdigitated microelectrodes, and a detection limit of 0.0128 HAU was achieved. For the detection of norovirus [109], Chand and Neethirajan utilized a microfluidic chip integrated with a graphene-GNP composite deposited on an SPCE. This nanocomposite of graphene-GNP provided a better substrate for immobilization and enabled excellent electron transfer at the interface. The interaction between norovirus and the ferrocene-labelled aptamer resulted in a reduced electrochemical signal when the nanocomposite was coated onto the, resulting in a norovirus detection range of 100 pM to 3.5 nM.

Another group of researchers [110] described the application of optical transduction techniques for semi-quantitative analysis in order to detect the p24 antigen using a fully integrated multicolour immunosensor. This system simultaneously combined two-step reactions in a single microfluidic chip to achieve the horseradish peroxidase (HRP)-linked immunoassay and GNR-based multicolour assay. This sensor had a qualitative visual detection limit of 0.5 ng/mL, as well as high sensitivity and low serum protein interference. Other researchers used carbon dots with fluorescent labels that have a high quantum yield and low toxicity. Chunduri et al. [24] developed a microfluidic carbon dot sandwich immunoassay with a detection range of 20–1000 pg/mL, providing a 100-fold increase in detection over traditional colorimetric assays. This technique could be multiplexed to detect other pathogens, such as those causing tuberculosis and hepatitis. A POC device with a broad linear range and high specificity, selectivity, and sensitivity was created by Kaminska et al. [64] by combining SERS and microfluidics for the detection of the hepatitis virus. In their study, antibodies (anti-HBsAg) covalently linked to fuchsin (Raman reporter)-labelled gold nanoflowers served as the biosensing element, and the substrate was made up of Au–Ag-coated gallium nitride (GaN). The detection limit for HBsAg antigens could be increased to 0.625 IU/mL. In addition, the presence of antibodies against the SARS-CoV-2 spike protein was detected in diluted human plasma using an opto-microfluidic sensing platform based on LSPR developed by Funari et al. [30]. The electrodeposition technique was used to fabricate a substrate with gold nanospikes. The local refractive index shifts in the gold nanospikes caused by biomolecule binding events cause a redshift proportional to the target antibody concentration.

Suthanthiraraj et al. [22] exploited the silver nanostructure-based LSPR effect to detect dengue NS1 antigens in whole blood samples. These nanostructures were formed through the thermal annealing of thin silver films onto a silicon substrate at 200 °C for 1 h. Silver nanostructures were chosen for this study because of their intrinsic ability to produce huge wavelength shifts and the wide differences in the real part of the dielectric constant within the visible range. The sensor substrate was coupled with a blood plasma separation platform to create a lab-on-chip device for quick detection, providing a sensitivity of 9 nm/(g/mL). In another study, the human angiotensin-converting enzyme 2 protein was used as a biorecognition element in an LSPR-based sensor with AgNT [36]. It was tested against the SARS-CoV-2 spike RBD protein and CoV NL63 virus and could detect concentrations as low as 0.83 pM and 391 pfu/mL, respectively. The list of nanomaterials employed in the recent development of microfluidic technique-based virus detection is illustrated in Table 3.

**Table 3:** Microfluidic based detection of various viruses using nanomaterials

Nanomaterial	Nanomaterial size	Analyte	Biorecognition element	Method employed	Samples	Linear range	Limit of detection	Detection range	Label type	References
Graphene-GNP composite	GNP-16 nm	Norovirus	Norovirus viral capsid-specific aptamer	Electrochemical	Spiked blood samples	–	100 pM	100 pM to 3.5 nM	Label free	[109]
Gold nanoflowers/Au–Ag coated GaN	20–40 nm/90 nm	Hepatitis B virus	Anti-HBs Ag	SERS	Human blood plasma	0.0125–60 IU/mL	0.01 IU/mL	0–625 IU/mL	Label	[64]
Gold spikes	–	SARS-CoV-2	Anti-SARS-CoV-2 spike protein antibodies	LSPR	Plasma samples	–	0.08 ng/mL	0.1–10000 ng/mL	Label free	[30]
Silver nanostructures	20–80 nm	Dengue NS1	Anti-NS1 antibody	LSPR	Whole blood spiked with NS1 antigen	–	0.06 µg/mL	0–50 µg/mL	Label free	[22]
Silver nanotriangle	Side length-152 ± 3 nm height-60 ± 1 nm	SARS-CoV-2	Human angiotensin-converting enzyme 2 protein	LSPR	SARS-CoV-2 spike RBD protein and CoV NL63 virus	2.03 pM to 9420 pM	0.83 pM, 391 pfu/mL	1 pM to 10^4^ pM	Label free	[36]

*GNP* Gold nanoparticles, *SERS* Surface enhanced Raman scattering, *Au* Gold, *Ag* Silver, *Hbs* Hepatitis B, *SARS-CoV-2* Severe acute respiratory syndrome corona virus-2, *LSPR* Localised surface plasmon resonance, *NS1* Non-structural protein 1

### Nanopore-based biosensors in viral diagnostics

Mc Mullen and colleagues [111] described the primarily voltage-independent passing of a stiff filamentous virus (880 nm long and 6.6 nm wide) through a solid-state membrane. The Filoviridae Marburg and Ebola viruses fulfil these requirements. While the current was not the driving force, the electric field helped align the virus to the nanopore. Similarly, the translocation of a rigid rod-shaped virus, a tobacco mosaic virus, through a solid-state nanopore was described in a paper by Wu et al. [112]. This study reported methods to determine the rigidity of (bio)material in general using solid-state nanopores. In a recent paper, data analysis was applied in combination with solid state nanopore analysis [113]. The authors highlighted the potential of solid-state nanopores in viral diagnostics through the detection and quantification of whole virus particles.

In addition to whole viruses, viral biomarkers have also been examined, and single-molecule detection of the nucleocapsid protein 7 (NCp7) from the HIV-1 virus has been performed using synthetic nanopores, specific aptamer–protein interactions, and the resistive-pulse technique [114]. Surface modification with solid-state nanopores has been applied to selectively detect viruses by exploiting the specific molecular interactions between recognition probes on the surface and target particles. This strategy has been adopted for selective single-virus identification [115]. Hemagglutinin antibody-mimicking oligopeptides with a weak affinity to influenza A virus have been utilized for functionalizing the pore wall surface, thus enabling specific virus–nanopore interactions. Such viral ligand binding led to an alteration in viral translocation dynamics in the nanochannel. Such a modification approach allowed the flexible introduction of specific interactions, and thus, the recognition and differentiation of distinct viruses and bacteria too. Along these lines, Taniguchi et al. [116] found that, by using nanopores in combination with artificial intelligence, coronaviruses of similar size could be identified. This could allow discrimination among coronaviruses such as HCoV-229E, SARS-CoV, MERS-CoV, and SARS-CoV-2. Moreover, this technique enabled the successful detection of SARS-CoV-2 in saliva. Together, solid-state virus immunosensing techniques hold promise in providing versatile, flexible, and cost-effective diagnostic tools in the future, particularly in combination with sophisticated data analysis, microfluidics, and chemical ingenuity.

### Internet of medical things and artificial intelligence in viral biosensors

The Internet of Things (IoT) is a rapidly evolving interdisciplinary domain that integrates computer hardware, software, other electronic devices, and physical objects, both living and non-living, across a network. Numerous IoT-enabled services are being experimented with in healthcare, connecting patients to healthcare units remotely [117]. This IoT network, known as the Internet of Medical Things (IoMT), is a rapidly evolving area in which patients and doctors are communicating with each other through a series of medical devices with wireless connectivity [118]. A rapid and accurate screening of health conditions represents a key step in order to identify the sign of symptoms of an infection disease although its effect did not come forward. However, the real-time identification of viral infections is difficult and expensive to manage within traditional healthcare settings due to their high response time, the need for qualified personnel, as well as the cost per analysis. In view of this consideration, the healthcare industry is striving toward extending the accuracy, reliability, and productivity of biosensor-based POC devices. These diagnostic tools integrated with IoMT capacities can help in the screening, providing effective medical treatment, and most importantly, offers control over epidemic outbreaks [119, 120].

**Fig. 5:**
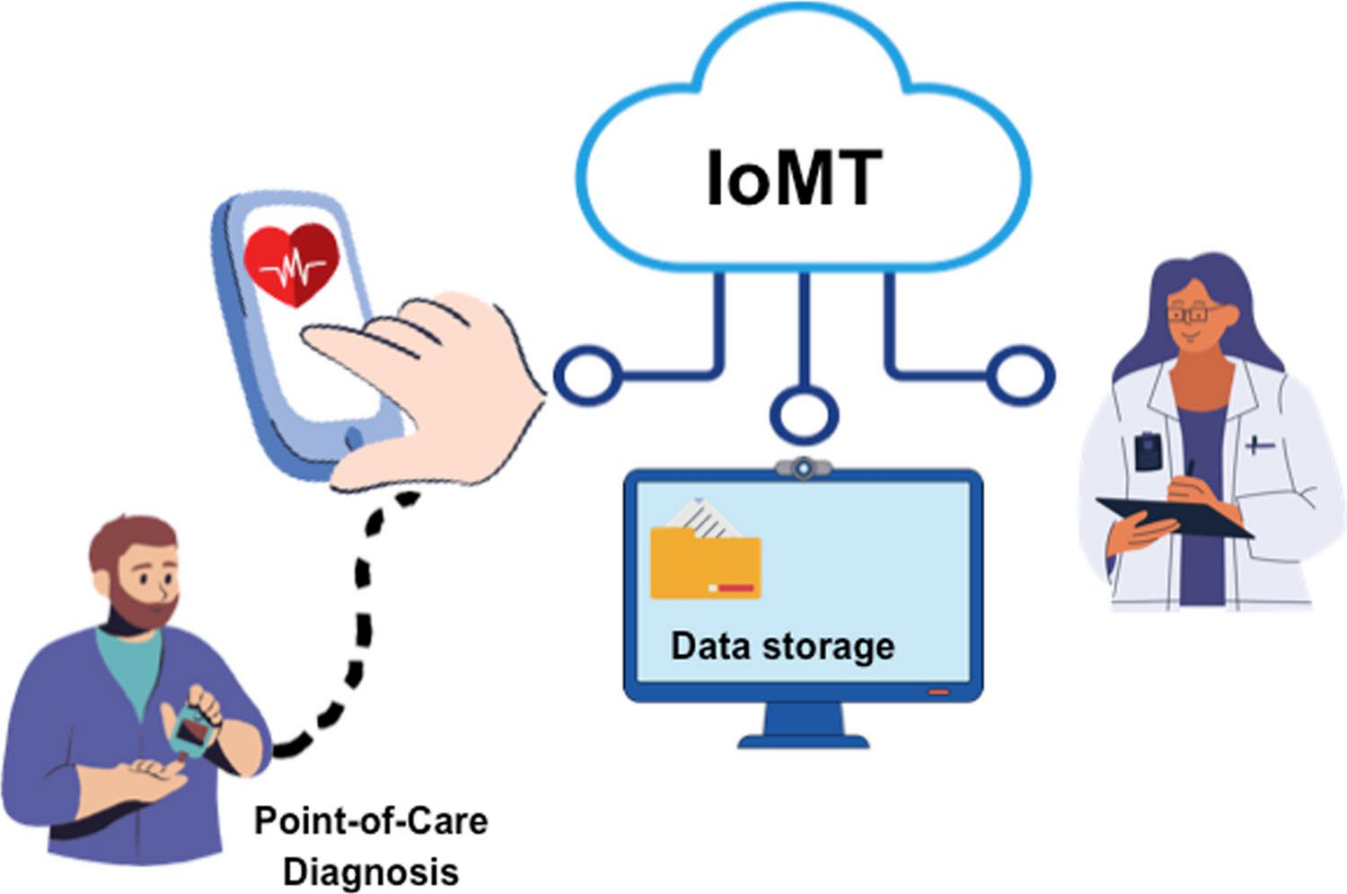
Scheme of Internet of Medical Things combined with smartphone-based platforms for POC diagnostic applications

Smartphones have a wide range of applications for biosensors due to their popularity, portability, versatility and wireless communication technology that allows the connection to cloud data storage systems and IoT networks for collecting and sharing medical analysis data [121] Fig. 5. Moreover, smartphone-based biosensors are attractive to researchers because they can easily enable both qualitative and quantitative analysis of the sample in real time using a smartphone application that can perform the measurement of colorimetric, fluorescent, reflection-based, current, and turbidity signals [122, 123]. They are able to control the recognition process, receive recognition data through various interfaces such as Bluetooth, camera, audio jack, and micro-USB port, and display recognition results [124, 125]. Therefore, these features make smartphone-based devices ideal for developing diagnostics for virus detection that can be performed outside of the clinical laboratories.

An OFF–ON biosensors for detection of H1N1 based on the interaction of QD-aptamer beacons with three-dimensional light guides photonic crystal was proposed from Nuree Lee et al. [126]. High sensitivity with LOD of 138 pg and high mL^−1^ and selectivity over other species of influenza A virus and biomolecules was obtained. The target virus was detected with a low-cost and portable home-made setup coupled with smartphone.

Recently, paper-based biosensors coupled to smartphones as transducers for rapid virus detection have gained much attention as a useful tool for rapid, easy, affordable, and accurate POC. Teengam et al. [127] developed paper-based analytical device for the early diagnosis of HCV using a highly specific pyrrolidinyl peptide nucleic acid (acpcPNA) probe. The acpcPNA was covalently immobilized onto oxidized cellulose paper via reductive alkylation between the aldehyde and amine groups. The detection occurred by monitoring the fluorescence signal response of a fluorescent dye that selectively binds the single-strand region of the DNA target over the PNA probe using a smartphone camera. The obtained LOD was 5 pmol^−1^.

An interesting miniaturized paper-based smartphone immunosensor for differential diagnosis of wild-type pseudorabies virus (PRV) infection versus vaccination immunization was proposed by Yong Tang et al. Latex beads were used to label PRV, and the test line (T-line) was coated with PRV gE monoclonal antibody (PRV gE-mAb). The smartphone was used to measure the transmitted light intensity from the T-line achieving excellent sensitivity and selectivity [128].

Although smartphones perform the same data collection, processing and decision tasks like a computer, they have some limitations. First, functionality of each smartphone app is independent of the other apps. In addition, more attention should be given to data security because the smartphones hold personal information.

**Fig. 6:**
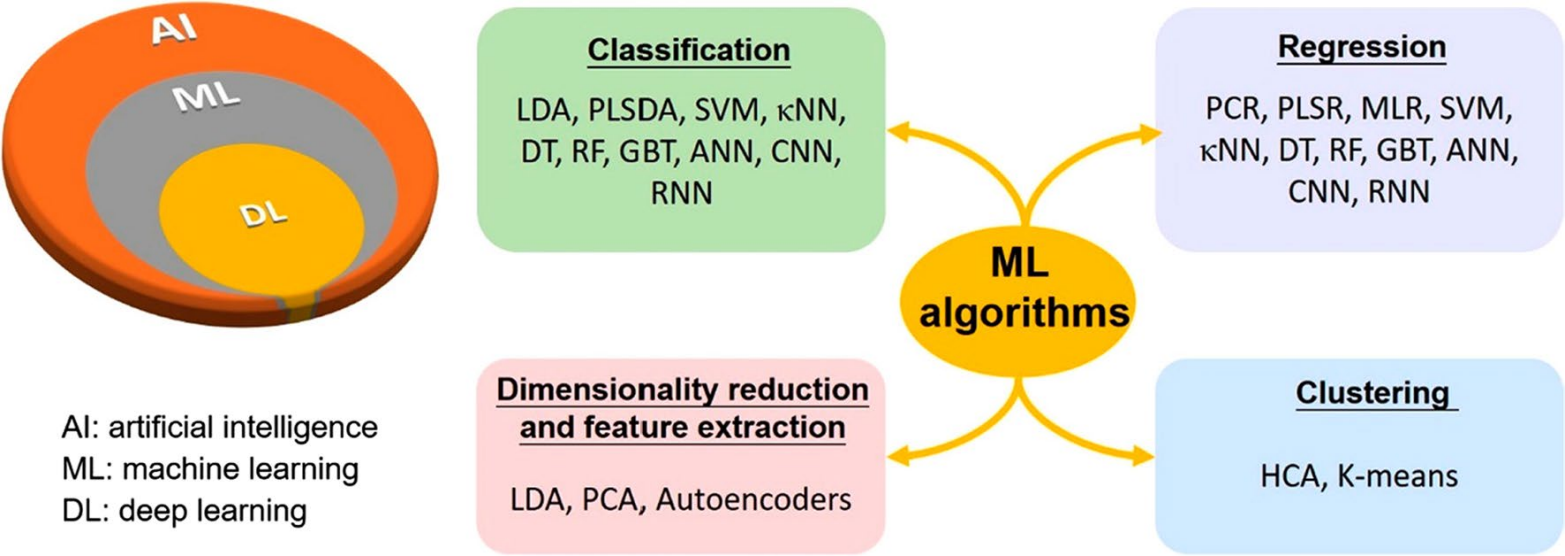
Relationship between AI, ML, and DL, and various ML algorithms applied to Biosensors. *CNN* Convolutional neural network; *DT* decision tree; *Feedforward ANN* Feedforward artificial neural network; *GBT* Gradient-boosted trees; *HCA* Hierarchical clustering analysis; *κNN* κ-nearest neighbour; *LDA* Linear discriminant analysis; *MLR* Multiple linear regression; *NB* Naive Bayes; *PCA or PCR* Principal component analysis or regression; *PLSDA or PLSR* Partial least-squares discriminant analysis or regression; *RF* Random forest; *RNN* Recurrent neural network; *SVM* Support vector machine. Reprinted with permission from [129]. Copyright {2020} American Chemical Society

In some biosensors, a large quantity of data is rapidly generated as output. The analysis of these data requires additional processing by an experienced user, making the process error prone. Moreover, manual data processing is time-consuming, greatly reducing the efficiency of the biosensor. In the past years, numerous chemometric methods such as principal component analysis or regression (PCA or PCR), linear discriminant analysis (LDA), multiple linear regression (MLR), partial least-squares discriminant analysis or regression (PLSDA or PLSR), hierarchical clustering analysis (HCA), and their combination were applied in the field of biosensor technology in order to improve the quality and accuracy of clinical information. In the last decade, an increasing number of studies in the field of AI and ML have indicated the growing interest and scope of its use in this area for their ability to interrogate appropriate nonlinear dependencies for complex biological samples, offering unparalleled opportunities to address pressing biosensors challenges. ML algorithms for processing data are emerging, such as κ-nearest neighbour (κNN), support vector machine (SVM), Naïve Bayes (NB), decision tree (DT), gradient-boosted trees (GBT), random forest (RF), feedforward artificial neural network (Feedforward ANN), recurrent neural network (RNN) and convolutional neural network (CNN) are investigated to create the intelligent biosensors that can be easily integrated into the IoMT (Fig. 6) [129–132]. AI-integrated biosensors combine hybrid techniques of wireless biosensing technology and advanced ML algorithms, holding great promise for realizing continuous health monitoring and cloud-connected POC diagnostics [133]. They can be of great importance in the prediction of infectious diseases in which early interventions can be lifesaving (Table 4). They can bridge the gap between data acquisition and analysis and achieve enhanced diagnostic and therapeutic accuracy [134]. Despite the limited number of commercial applications, continuous remote sensing using wearable such as glucose monitoring devices, oximeters, temperature sensors, and heart and respiratory rate monitors connected to an AI-based central computing device is revolutionizing patient care. In addition, ML can be a possible solution to overcome the challenges and obstacles faced by biosensor testing in complex samples [135]. Most biosensors were developed for target detection using body fluids such as blood or urine samples. Recently, the application of additional biological matrices such as nasopharyngeal swab, oral secretions and body sweat has increased significantly because the tests are non-invasive, safe, pain-free, and easily operable. However, many physiological and pathological factors can influence the composition of these body samples production, including age, drug use, hormonal conditions, psychological stature, and physical recreation. ML can support biosensors by increasing their reliability, accuracy (objective identification), specificity (pattern recognition) and sensitivity (single molecule detect).

**Table 4:** List of some AI-biosensors for viral diseases detection

Device	Detection principle	Target	Limit of detection	Time (min)	References
*Dengue fever*					
NAAT	QUASR multiplexed RT-LAMP assay	RNA	10^8^–10^3 ^pfu/mL	< 40 min	[164]
IoT PCR	PCR	cDNA	–	34 min	[165]
*Influenza A*					
Fluorescent probe based POC	Fluorescence and light guiding	H1N1	138 pg/mL	40 min	[126]
Genosensor	Electrochemical	Hemagglutinin gene	0.002 ng/6 μL	30 min	[166]
*Human immunodeficiency virus*					
EGOFET	Electrochemical	HIV-1 p24 capsid protein	1 fM	–	[167]
*Coronavirus-2*					
Supersandwich-type biosensor	Electrochemical	SARS-CoV-2 RNA	200 copies/mL	–	[168]
Liquid crystal film	Thermotropic Liquid	RNA-CoV2	fM	20 min	[169]
Sensors	Localized Surface Plasmon Resonance	SARS-CoV-2 virus particle	125.28 vp/mL		
*Others*					
Plasmon resonance probes	Dark-field microscope	Enterovirus A71	3 copies/μL		[132]
Immunochromatographic strip	LFA	EBOV-GP_1−649_	200 ng/mL	15 min	[170]
Impedimetric micro-immunosensor	Electrochemical	ZIKV protein	10 pM	40 min	[171]

*cDNA* Complementary deoxyribonucleic acid, *NAAT* Nucleic acid amplification tests, *QUASR* Quenching of unincorporated amplification signal reporters, *EGOFET* Electrolyte-gated organic field-effect transistor, *ELISA* Enzyme-linked immunosorbent assay, *LAMP* Loop-mediated isothermal amplification, *LFA* Lateral flow assay, *LFD* Lateral flow dipstick, *MCFA* Microchannel capillary flow assay, *RNA* Ribonucleic acid, *RPA* Recombinase polymerase amplification, *RT-LAMP* Reverse transcription loop-mediated isothermal amplification, *SARS* Severe acute respiratory syndrome

During the COVID-19 pandemic, the IoMT, applications of AI and ML in POCs have drastically grown [136]. Due to the contagious nature of the virus governments-imposed lockdowns on cities to prevent the virus from spreading. Unfortunately, acquiring medical supplies made it necessary to come out of isolation, which compromised the effectiveness of quarantine efforts. Better outcomes for controlling the spread of infection and offering adequate healthcare to patients without putting others at risk, it was POCs use to monitor patient health at home and IoMT creation to share information with doctors and hospitals [136–138]. Biosensors have been incorporated into simple miniaturized analytical devices that enable SARSCoV-2 diagnosis, monitoring, and management with various analytical approaches [139–141].

A portable smartphone-based quantum barcode serological assay device for real-time surveillance of patients infected with SARS-CoV-2 was developed by Chan et al. Instantaneous results to inform patients, physicians, and public health agencies was obtained by appropriate app [142]. A liquid crystal-based diagnostic kit and a smartphone-based application to enable automatic detection of SARS-CoV-2 ssRNA was realized. The analytical tool was applied for reliable self-test at femtomolar concentrations of single-stranded ribonucleic acid (ssRNA) SARS-CoV-2 [143].

A Nanozyme linked immunochromatographic sensor integrated with optical devices for the detection of SARS-CoV-2 nucleocapsid protein has been developed. Immunoreaction and enzyme-catalyzed substrate colour reaction were carried out on the chromatographic strip in a device, of which the light signal was read by a photometer through a biosensor channel, and the data was synchronously transmitted via the Bluetooth to the app in-stored smartphone for reporting the result. A LOD of 0.026 ng/mL was achieved [144].

**Fig. 7:**
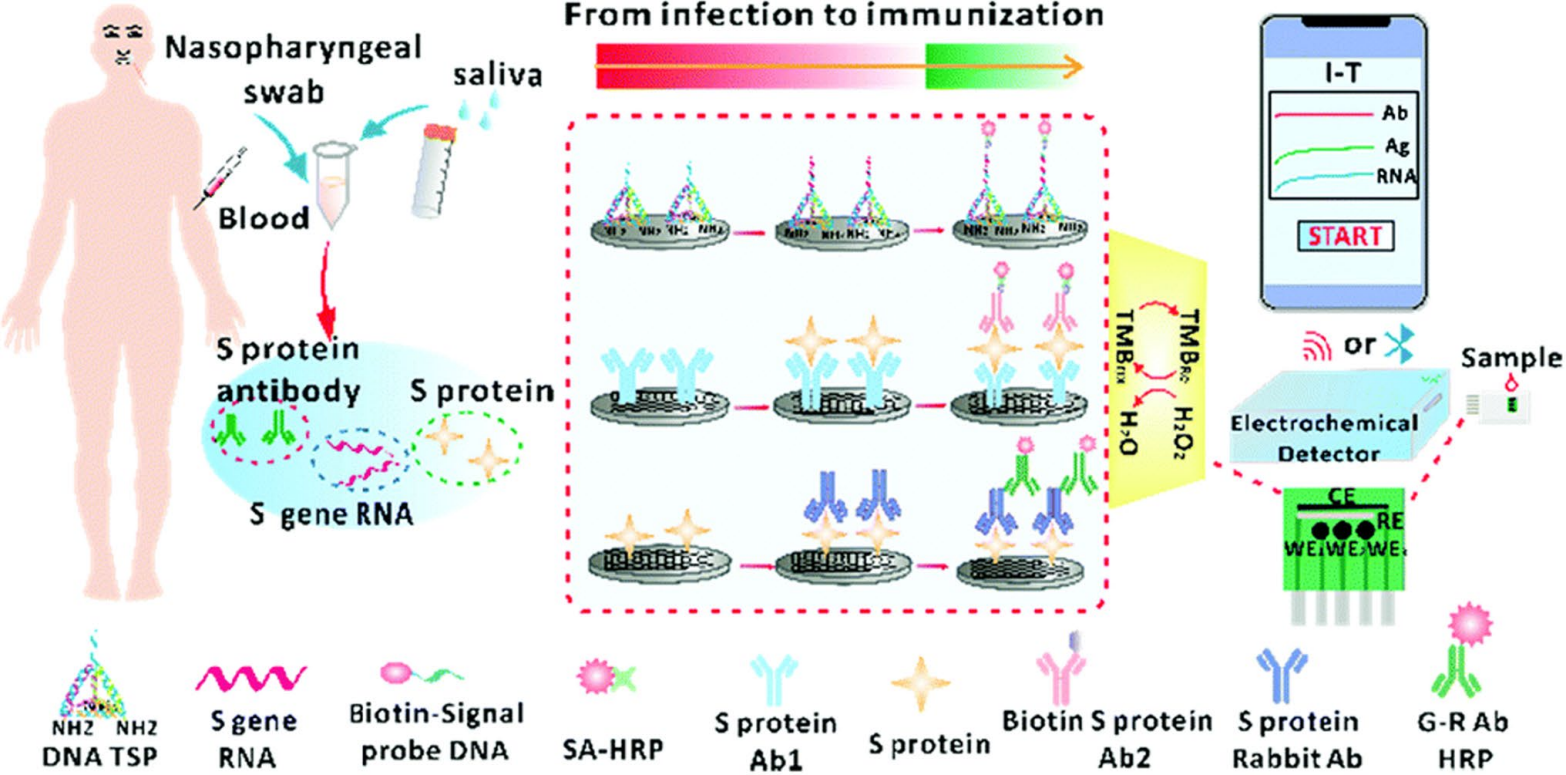
Scheme of electrochemical co-detection procedure for S gene RNA, S protein and S protein antibody of SARS-CoV-2 by sandwich approach. The biosensor is composed of three testing parts, each of which consists of a carbon working electrode (WE), a Ag/AgCl reference electrode (RE), and a carbon counter electrode (CE). Selective binding of different concentrations of SARS-CoV-2 biomarkers to surface-linked capture probes should produce the current change when the specific biomarker was present. The harvested current information was transmitted to the smartphone via wireless connections. The special APP was used for data analysis. Reprinted Chem. Commun., 2022, 58, 6108 [145]

Multiplexing analysis has attracted researcher attention for clinical diagnosis because a number of diseases require analyse complex biological networks instead of analysing a single marker. Therefore, biosensors for multi-target analyses might facilitate infection screening and improve the rates of earlier detection and attendant improved prognosis were developed. Dou et al. designed a biosensor for co-detection of SARS-CoV-2 viral RNA, antigen, and antibody in combination with a smartphone. The online monitoring of SARS-CoV-2 virus-infected patients from infection to immunization was accomplished (Fig. 7) [145].

The multiassay was applied in saliva samples for the detection of SARS-CoV-2. To increase the biosensors sensitivity, a paper-based immunoassay using magnetic beads to support the immune chain and 96-well wax-printed paper plate for colour background smartphone viewing in conjunction with the free-charge app was developed; a LOD of 0.1 μg/mL was obtained [146].

A multiplexed, colorimetric detection method was established for the detection of pathogens in wastewater samples to provide early warning of disease outbreaks. The developed method integrates on-chip nucleic acid extraction, two-step isothermal amplification, and colorimetric detection on a 3D-printed microfluidic chip. Colorimetric signals during nucleic acid amplification were recorded in real time and analysed by a programmed smartphone. The device exhibited potential for rapid spatiotemporal epidemiologic data collection regarding the environmental dynamics, transmission, and persistence of infectious diseases [147].

AI and ML biosensors are applied for detecting hidden disease signatures and for the management of COVID-19 [67, 140, 148–155]. It is known that early symptoms of SARS-CoV-2 infection overlap with other common conditions such as common cold and Influenza, making early screening and diagnosis are crucial goals for health practitioners. The parameters of the full blood counts can be analysed to distinguish the viral type at an earlier stage. Abhirup Banerjee et al. [150] successfully applied ML, artificial neural network and a simple statistical test to identify SARS-CoV-2 positive patients from full blood counts without knowledge of symptoms or history of the individuals. Moreover, AI is transforming medical practice and precision medicine in intensive care unit Safe, effective, efficient, and ethical clinical management of COVID-19 patients [151].

Despite efforts to identify proper new smart biosensors for intelligent healthcare based on the synergic integration of the IoMT, ML and AI for rapid and efficient selective SARS-CoV-2 diagnosis, research remains in its nascent stage. There is a clear need to increase highly sensitive non-invasive home-based diagnostic tools that mitigate testing and monitoring problems. By combining such devices with the IoMT and AI, a smart platform that provides timely patient care protocols could be developed and could help governmental organizations suitably allocate the apparatus and other necessities for viral disease management. This approach could help save numerous lives, provide economic benefits, and develop a plan for better managing the future risks caused by infectious diseases, conferring benefits across all levels of society.

## Challenges and future perspectives

It is expected that commercially viable diagnostic tools based on nanomaterials that efficiently and cost-effectively perform multiplexed detection will become available in the foreseeable future. The miniaturisation of nanochip technology is going to be the future trend in diagnostics. The long-term outlook for nanobiosensors in disease diagnosis could result in high-volume sales and rapid adoption. However, there are some challenges associated with using biosensors today, such as the transformation of nanobiosensors from prototypes into commercial products. This is because field-scale trials are necessary to gauge and evaluate the effectiveness of nanobiosensors in practical settings, and user awareness of nanobiosensors is also crucial. End users may face real-time problems due to the integration and interrelationships of the numerous technologies involved in nano-diagnostics. They may expect minimally invasive procedures for sample collection and/or avoidance of liquid biopsy. In order to accomplish this, a nanoneedle could be used, and these are slowly advancing towards clinical adoption [156]. Simple nano patches that can be applied to the skin’s superficial surface could be developed as advanced tools for detection and diagnosis based on interstitial fluids exudated through skin pores. Additionally, the nanobiosensors developed ought to be biodegradable to prevent any environmental harm [157, 158]. Overall, the integration of wearable biosensors [159] and IoMT [160] along with nanotechnology could have a significant impact on healthcare in the future.

Currently, a few companies, including Roche, Nippon, Robert Bosch, and IBM, appear to be working towards the fabrication of nanobiosensors. Reports on commercial nanobiosensors are limited. Although there are some reports of commercial nanobiosensors being used in medical diagnostics and applications, there are still some limitations beginning with the high cost of fabrication, the requirement of automated testing, rapid result evaluation, and field trial validation, which forces the miniaturization of prototypes into the industry for production. Additionally, there is no market to compensate for and cover all of these costs. That could partly explain the dearth of commercial nanobiosensors. However, obtaining novel nanomaterials from leftover biomass could provide a more affordable option [161]. The versatility of nanobiosensors is another issue that requires more attention. More portable nanobiosensors may become commercially viable if a variety of nanomaterials for biosensing assays are developed [162]. In the future, nanotechnologies could represent a promising solution for molecular diagnostics, enabling POC diagnosis, and also for theranostics and the development of personalized medicine.

## Conclusion

Nanotechnology has played a key role in the development of sensors for biomedical applications. Advances in nanomaterial synthesis and nanofabrication techniques have facilitated the development of innovative nanobiosensors for viral diagnostics. Integrative approaches for nanomaterial-based biosensors are vital, as traditional viral detection methods are time-consuming and expensive. Despite the range of their applications, nanobiosensors possess their own limitations, such as challenges in fabrication, sample preparation, substrate formation, bioreceptor immobilization, and appropriate substrate functionalization. These challenges must be overcome because they directly affect the sensitivity, reproducibility, detection limit, and selectivity of the nanobiosensors. The proper optimization of these parameters along with automation can significantly impact the development of highly sensitive nanobiosensors. This is an exciting time to be involved in the development of nanobiosensors, which should be designed in such a way that they are disposable and self-contained, allow remote sensing, and act as part of fully autonomous systems with interconnected sample processing and analysis in resource-constrained environments. Despite the great challenges, there are great expectations ahead.

## Abbreviations

3D: Three-dimensional
Ab: Antibody
Ag: Silver
AgNPs: Silver nanoparticles
AgNTs: Silver nanotriangles
AI: Artificial intelligence
Au: Gold
CCTS: Cu_2_CdSnS_4_
cDNA: Complementary deoxyribonucleic acid
CNF: Carbon nanofiber
CNT: Carbon nanotubes
COVID-19: Coronavirus disease
DENV: Dengue virus
DNA: Deoxyribonucleic acid
EGOFET: Electrolyte-gated organic field-effect transistor
ELISA: Enzyme-linked immunosorbent assay
GaN: Gallium nitride
GCDE: Glassy carbon disc electrodes
GCE: Glassy carbon electrode
GE: Glass electrode
GNP: Gold nanoparticle
GNR: Gold nanorod
GO: Graphene oxide
H1N1: Influenza A
HAU: Hemagglutination unit
Hbs: Hepatitis B
HBV: Hepatitis B virus
HCV: Hepatitis C virus
HEV: Hepatitis E virus
HIV: Human immunodeficiency virus
HPV: Human papilloma virus
HRP: Horseradish peroxidase
IDE: Interdigitated electrode
IoMT: Internet of medical things
IoT: Internet of things
LAMP: Loop-mediated isothermal amplification
LFA: Lateral flow assay
LFD: Lateral flow dipstick
LSPR: Localised surface plasmon resonance
MCFA: Microchannel capillary flow assay
MERS: Middle East respiratory syndrome coronavirus
MERS-CoV: Middle East respiratory syndrome coronavirus
ML: Machine learning
MWCNT: Multi-walled carbon nanotubes
NAAT: Nucleic acid amplification tests
NCp7: Nucleocapsid protein 7
NP: Nanoparticles
NS1: Non-structural protein 1
PAMAM: Polyamidoamine
PLM: Polystyrene latex microspheres
POC: Point-of-care
PRV: Pseudorabies virus
QD: Quantum dots
QUASR: Quenching of unincorporated amplification signal reporters
RBD: Receptor binding domain
rGO: Reduced graphene oxide
RNA: Ribonucleic acid
RPA: Recombinase polymerase amplification
RSV: Respiratory syncytial virus
RT-LAMP: Reverse transcription loop-mediated isothermal amplification
SARS: Severe acute respiratory syndrome
SARS-CoV: Severe acute respiratory syndrome coronavirus
SARS-CoV-2: Severe acute respiratory syndrome corona virus-2
SERS: Surface-enhanced Raman scattering
SiO_2_: Silicon dioxide
SPE: Screen-printed electrodes
SPGE: Screen-printed gold electrodes
SPR: Surface plasmon resonance
ssDNA: Single-stranded DNA
TMB: Tetramethylbenzidine
ZnO: Zinc oxide
